# The Stability of a Stochastic CaMKII Switch: Dependence on the Number of Enzyme Molecules and Protein Turnover

**DOI:** 10.1371/journal.pbio.0030107

**Published:** 2005-03-29

**Authors:** Paul Miller, Anatol M Zhabotinsky, John E Lisman, Xiao-Jing Wang

**Affiliations:** **1**Volen Center for Complex Systems, Brandeis UniversityWaltham, MassachusettsUnited States of America; **2**Department of Physics, Brandeis UniversityWaltham, MassachusettsUnited States of America; **3**Department of Chemistry, Brandeis UniversityWaltham, MassachusettsUnited States of America; **4**Department of Biology, Brandeis UniversityWaltham, MassachusettsUnited States of America; Hebrew UniversityIsrael

## Abstract

Molecular switches have been implicated in the storage of information in biological systems. For small structures such as synapses, these switches are composed of only a few molecules and stochastic fluctuations are therefore of importance. Such fluctuations could potentially lead to spontaneous switch reset that would limit the lifetime of information storage. We have analyzed a model of the calcium/calmodulin-dependent protein kinase II (CaMKII) switch implicated in long-term memory in the nervous system. The bistability of this switch arises from autocatalytic autophosphorylation of CaMKII, a reaction that is countered by a saturable phosphatase-1-mediated dephosphorylation. We sought to understand the factors that control switch stability and to determine the functional relationship between stability and the number of molecules involved. Using Monte Carlo simulations, we found that the lifetime of states of the switch increase exponentially with the number of CaMKII holoenzymes. Switch stability requires a balance between the kinase and phosphatase rates, and the kinase rate must remain high relative to the rate of protein turnover. Thus, a critical limit on switch stability is set by the observed turnover rate (one per 30 h on average). Our computational results show that, depending on the timescale of fluctuations in enzyme numbers, for a switch composed of about 15 CaMKII holoenzymes, the stable persistent activation can span from a few years to a human lifetime.

## Abstract 2

Computational modeling indicates that autophosphorylation of CaMKII can create stable persistent activation lasting several years

## Introduction

Molecular switches have been implicated in many types of cell-biological processes including the storage of decisions about cell fate [1], genetic control [2], and memory storage in the brain [3]. The mechanisms of such switches generally depend on some kind of autocatalytic process. If a switch is composed of a small number of molecules, stochastic fluctuations are significant and a deterministic description is not sufficient [4]. Because of the dynamic interaction of opposing reactions, such fluctuations can spontaneously reset the state of a switch. Reset events of this kind impose a temporal limit on the usefulness of the switch for information storage. It is thus crucial to understand the factors that control switch stability and to develop quantitative insight into how the stability required for a particular biological process could be achieved. The stability problem of switches has so far been studied primarily in relation to genetic switches [5,6,7,8,9].

The problem of switch stability is of particular relevance to synaptic function [10,11] since memory is thought to be encoded by changes in synaptic strength [12] and because there are indications that synaptic strength is controlled by molecular switches [13,14,15]. By a molecular switch, we mean a molecule or a small group of molecules that can undergo a persistent change in state. In our definition, the change in state occurs in a discrete rather than a smoothly graded way. Clearly, spontaneous reset of a synaptic switch that encodes memory would be problematic because it would lead to loss of the stored memory. The fact that at least some memories persist for a human lifetime indicates that storage processes of extraordinary stability are present.

The mechanisms that underlie synaptic information storage are beginning to be elucidated [16]. It has been demonstrated that brief periods of strong stimulation can lead to an increase in the strength of synapses, a process termed long-term potentiation (LTP) [17]. In vivo studies show that LTP can persist for at least a year [18]. The initiation of LTP is caused by activation of N-methyl-D-aspartate (NMDA) channels and elevation of intracellular calcium (Ca^2+^) concentration [19,20]. There is general agreement that the resulting activation of calcium/calmodulin-dependent protein kinase II (CaMKII) plays a critical role in LTP (reviewed in [3]). CaMKII activation is persistent [21], is required for LTP [22,23,24], and is sufficient by itself to produce potentiation [25]. Genetic modification of CaMKII that prevents its sustained activation prevents long-term memory, as defined in behavioral tests [24]. The possibility that CaMKII is a synaptic memory molecule is further strengthened by the finding that it has autocatalytic properties that would allow it to function as a molecular switch [10,26,27]. Although CaMKII is required for long-term synaptic modification and is therefore a strong candidate as a memory molecule, whether evidence that its persistent activation is necessary for the maintenance of LTP remains an open question [23].

In a previous analysis of the CaMKII switch, Zhabotinsky and Lisman [28,29] proposed a model that incorporated many key biochemical properties of CaMKII holoenzymes and the phosphatase-1 (PP1) enzymes that dephosphorylate them [30,31]. It was shown that an interplay between autophosphorylation of CaMKII holoenzymes and dephosphorylation by PP1 molecules can give rise to two stable states of phosphorylation at basal levels of free Ca^2+^. Therefore, a transient input of high Ca^2+^ (such as during the stimulation protocol used in LTP induction) can switch the system from an unphosphorylated (DOWN) state to a persistent, highly phosphorylated (UP) state. Such a persistent change in activation of CaMKII following LTP induction could underlie the persistent change in synaptic strength. In these previous modeling efforts, chemical reactions were described deterministically by the law of mass action, precluding estimates of the switch stability. The need for considering the limits on stability imposed by stochastic fluctuations is made more urgent by recent measurements showing that the number of CaMKII holoenzymes in the postsynaptic density (PSD) of a single synapse is relatively small [32]. For a typical PSD, there are about 30 holoenzymes [32]. In the absence of a theory that relates switch stability to the number of switch molecules, the implications of this finding are unclear. The current work addresses this issue using Monte Carlo simulations of the stochastic chemical reactions in the CaMKII/PP1 system. This approach allows us to estimate quantitatively the stability (lifetime) of a CaMKII switch and its dependence on the number of molecules. Our results thus provide new information about the potential for the CaMKII switch within the PSD to store long-term memories.

A second goal of our work is to analyze the impact of molecular turnover on switch stability. Because biological switches are themselves composed of molecules that are unstable, turnover must occur. Such turnover is likely to have a detrimental effect on switch stability [33]. However, turnover need not necessarily lead to switch reset since new molecules may adopt a state that is dependent on the state of the other molecules in the switch [33,34]. Specifically, when the CaMKII switch is in the UP state, the PP1 activity should be saturated. This saturation reduces the effectiveness of the phosphatase so that when a phosphorylated holoenyzme is replaced by a newly synthesized unphosphorylated one, the new holoenzyme will become phosphorylated as a result of the autophosphorylation even at basal Ca^2+^ levels. This can restore the state of the switch that was present before the turnover event. Direct measurements show that the CaMKII at synapses turns over about once per day [35], a timescale much shorter than synaptic memory. However, no theory has been developed for any type of molecular switch that allows an estimation of how this turnover quantitatively affects stability. Here we examine this issue with regard to the CaMKII switch. Our findings reveal general principles with implications for other kinds of molecular switches.

## Results

### Autocatalysis Leads to Bistability

To understand the effect of stochastic fluctuations in molecular switches, we have implemented simulations of the CaMKII/PP1 switch model [28,29]. In this implementation, reactions are modeled stochastically using Monte Carlo methods and the number of CaMKII and PP1 molecules that are individually considered is comparable to the numbers contained within the PSD at single synapses.

A CaMKII holoenzyme is composed of two rings, each with six kinase subunits. Each subunit has a single phosphorylation site at Thr286/287 that, when phosphorylated, makes the subunit active even when Ca^2+^/calmodulin is no longer bound. Autophosphorylation of the site on a given “substrate” subunit proceeds if two necessary conditions are fulfilled [36]. Ca^2+^/calmodulin must bind to the “substrate” subunit in order to reveal its Thr286/287 site. Also, the counterclockwise neighboring “catalyst” subunit must be active. Hence, the initial autophosphorylation necessary to switch a ring “on” requires the binding of two molecules of Ca^2+^/calmodulin. Subsequent phosphorylation of other subunits within a ring is faster (see Figure 1A) since the phosphorylated subunit is constitutively active without Ca^2+^/calmodulin. Thus, only a single Ca^2+^/calmodulin is required to phosphorylate a “substrate” subunit if its counterclockwise “catalyst” neighbor is already phosphorylated. (Note that our results are unaffected by the direction of autophosphorylation, but based on geometric considerations, we assume it is asymmetric [27,37].) At the resting Ca^2+^ concentration, with our standard parameters, the initial autophosphorylation occurs at an average rate of one per 3.5 h per unphosphorylated ring, while the further phosphorylation steps occur at approximately one per 4 min per available “substrate” subunit. We assume that the molecules of PP1 held in the PSD can dephosphorylate any of the sites on any of the holoenzymes in the PSD. Furthermore, PP1 becomes saturated when the kinase becomes hyperphosphorylated [29].

**Figure 1:**
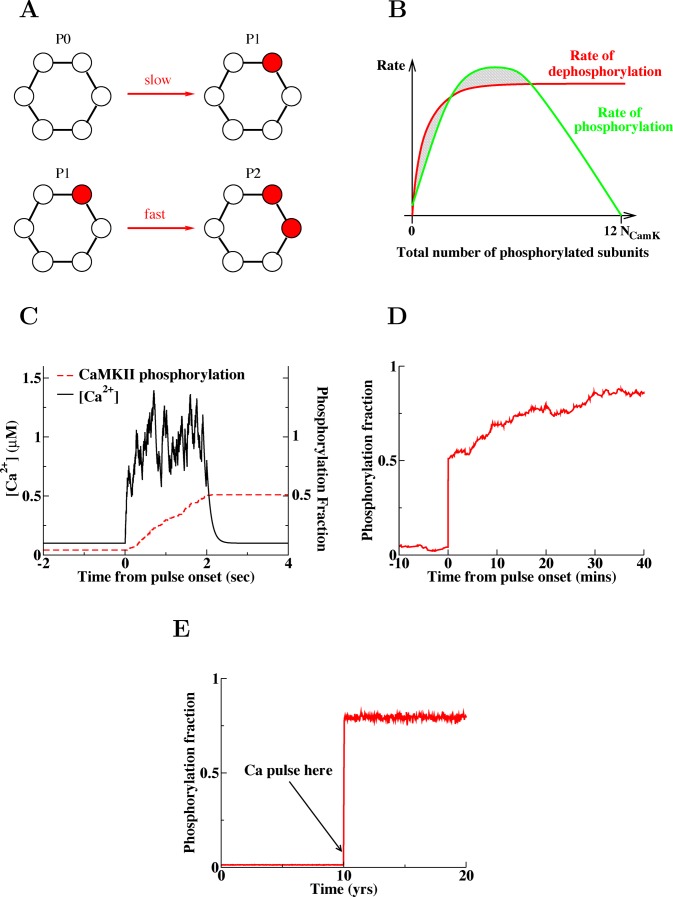
CaMKII Autophosphorylation and PP1 Saturation Lead to Bistability in the Phosphorylation States of CaMKII at Resting Ca^2+^ (A) Phosphorylation of the first subunit is slow at resting Ca^2+^ because of the requirement for two Ca^2+^/calmodulin molecules (see text). Subsequent phosphorylation is faster because only one Ca^2+^/calmodulin is required. (B) Schematic figure indicating how bistability arises from the dependence of phosphorylation and dephosphorylation rates on the number of subunits phosphorylated. Stable states are at the left (DOWN) and right (UP) intersections of the two curves. The middle crossing is unstable. The greater the area of the shaded region between a stable steady state and the unstable steady state, the harder it is for fluctuations to destabilize that stable steady state (the larger their basins of attraction). (C) A 2-s pulse of high Ca^2+^ switches the system (with 16 holoenzymes) from a low state of phosphorylation to a higher state within the basin of attraction of the UP state (see [D]). Phosphorylation fraction is *Sp_tot_*/12*N_CaMK_*. (D) After the end of the Ca^2+^ pulse, it can take tens of minutes for the system to reach the dynamic equilibrium in the UP state. (E) The UP state is stable for many years in a system with 16 holoenzymes.

Figure 1B indicates schematically how the total rates of autophosphorylation and dephosphorylation lead to two stable states at the resting Ca^2+^ level. The curves in the figure show how these reactions vary as a function of the total number of sites phosphorylated. The intersection points (where dephosphorylation balances phosphorylation) define three steady states, of which the left and right ones are stable and the middle one is unstable. Switching can occur if phosphorylation of the system is forced (either by a transient signal or by a spontaneous fluctuation) far enough away from one stable value that it passes the unstable value; the system will then fall into the basin of attraction of the second stable point. Once in this basin, the intrinsic dynamics of the system set the timescale of drift to the second stable state.

The model presented here includes stochastic turnover of CaMKII holoenzymes with an average lifetime of 30 h that is independent of phosphorylation level, as experimentally determined [35]. We assume, except where stated otherwise, that once a holoenzyme is removed, it is immediately replaced by an unphosphorylated holoenzyme. If the switch is in the DOWN state, the newly inserted holoenzyme is likely to stay off: any spontaneous phosphorylation will be countered by dephosphorylation, which removes a subunit in approximately 5 min, on average. However, if the switch is in the UP state, the phosphatase is so saturated by other phosphorylated holoenzymes, that the average time for a newly inserted subunit to be dephosphorylated is almost 1 h. Hence, the newly inserted holoenzyme can turn on as a result of the activation of the kinase by basal Ca^2+^ levels [28,29], since the time for it to be phosphorylated is significantly less than the time for turnover. Thus, the UP state of the switch is stable, in spite of turnover.

Parameters for the model were constrained according to the references cited in Table 1. Where the constraints allowed a range of variation, we chose values of parameters so that the system would be bistable and have approximately equal lifetimes of the UP and DOWN states (see below). We required that our standard system have equal numbers of CaMKII holoenzymes and PP1 molecules, as their concentrations are known to be similar, but adjusted the less well-determined Hill constants, *Κ_H1_* and *Κ_H2_,* to maximize system lifetime (see below). While our model is more sensitive to the value of *Κ_H1_* than any other parameter, bistability still exists in a significant range (10% around its optimal value) when all other parameters are fixed. Compensatory covariation of other parameters maintains the system's bistability at fixed calcium when *Κ_H1_* varies by more than a factor of three. Our study aims to test whether a plausible set of kinetic parameters enables a molecular switch, with a small number of participating molecules, to be stable in spite of fluctuations.

**Table 1:**
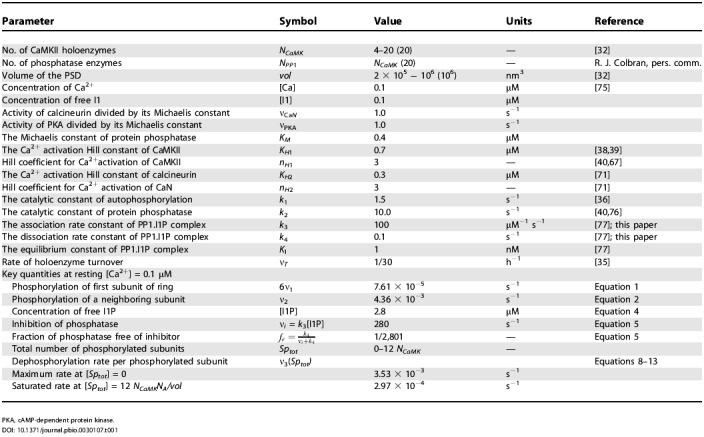
Parameters Used in the Model

PKA, cAMP-dependent protein kinase

Two critical requirements exist for the switch to function. First, the initial (P0 to P1) phosphorylation step must be significantly slower than further phosphorylation steps. This is true as the Hill constant for Ca^2+^ activation of CaMKII [38,39] is significantly greater than the average Ca^2+^ concentration in the physiological resting state (0.7 μM versus 0.1 μM in most of this paper). Second, the phosphatase activity must saturate so the rate of dephosphorylation per phosphorylated subunit is significantly slower in the UP state than in the DOWN state. This is achieved as the Michaelis constant, *Κ_M_,* of PP1 is much lower than the concentration of CaMKII subunits (0.4 μM versus 400 μM in this paper, though we also test the model with a *Κ_M_* of 10 μM [40]). Thus, according to the latest experimental data, the two critical requirements for a functioning switch are met.

Figure 1C and 1D (with 16 holoenzymes simulated) show that a large, 2-s-long Ca^2+^ elevation of the kind that may occur during LTP induction [41] can switch the system from the unphosphorylated DOWN state to a highly phosphorylated, persistent UP state. The system drifts toward the UP state even after Ca^2+^ falls to its basal level (Figure 1D). Such a drift has been observed experimentally [42]. During the drift period, which can take tens of minutes, the system would be more vulnerable to depotentiation. In this particular example (with 16 holoenzymes), the stable UP state is reached in under an hour (Figure 1D). As seen in Figure 1E, the resulting UP state remains stable for at least 10 y.

### Spontaneous Transitions between the Baseline and Memory States

We examined the distribution of times between spontaneous switching events as a measure of the stability of memory storage. Figure 2A shows that the total phosphorylation level of a small system with eight holoenzymes is only stable on the time scale of months, not years; sporadically, fluctuations cause the system to change from one state to the other, as indicated by the random switching of phosphorylation level. Analysis reveals that the times spent in either the DOWN or UP state (i.e., the lifetimes) are distributed exponentially (Figure 2B) (apart from brief transition times). Such an exponential distribution [43] indicates that the probability of transition per unit time is constant for a given state. The exponential distribution of lifetimes has a characteristic time constant that equals the average lifetime in the state (and the inverse of the probability of transition per unit time).

**Figure 2:**
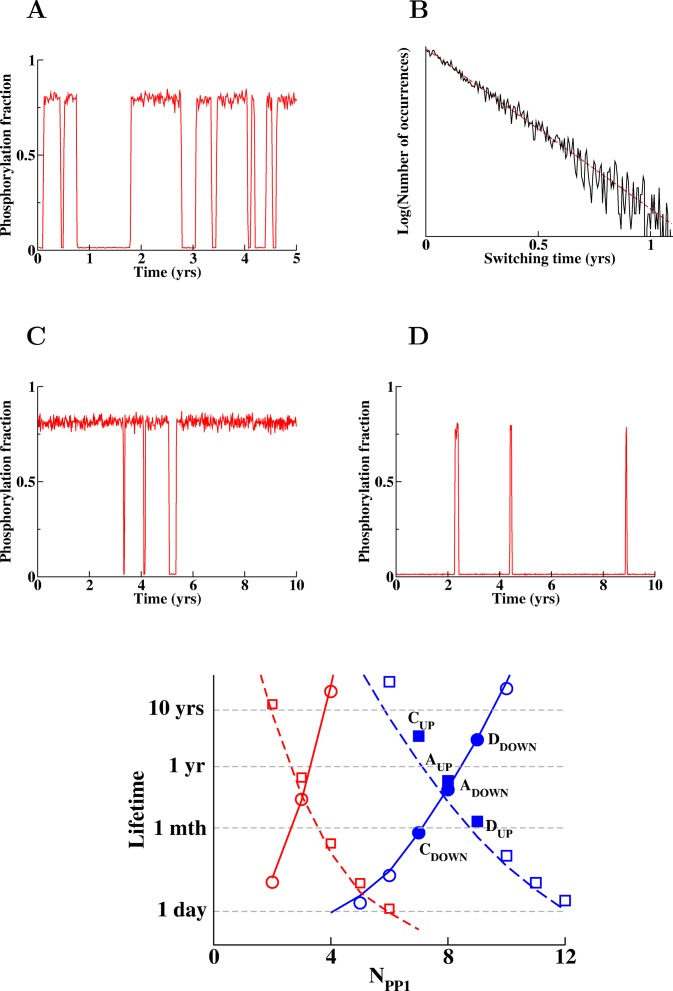
Switch Stability Is a Trade-Off between Lifetimes of UP and DOWN States (A) Spontaneous switching between UP and DOWN states in a system with eight CaMKII holoenzymes. (B) The distribution of lifetimes between switching events is exponential, as demonstrated by the straight line fit for lifetimes of the UP state on a semi-logarithmic scale. (C and D) Removing one PP1 enzyme (seven instead of eight) (C) leads to a longer lifetime for the UP state but shorter lifetime for the DOWN state; whereas adding one PP1 enzyme (nine instead of eight) (D) yields the opposite effect. (E) Dependence of average lifetimes of the UP state (squares) and DOWN state (circles) as a function of the number of PP1 enzymes (with eight CaMKII holoenzymes). Filled blue symbols correspond to data points from (A), (C), and (D). Lines are approximate, analytic results (based on Materials and Methods and [51]). The optimal lifetime of the switch is defined by the intersection point of the two curves, at which the lifetimes of the UP and DOWN states are equal. Blue indicates reference parameters. Red indicates *k*_1_ = 0.75 s^−1^, one-half of the standard value. The lifetime does depend on kinetic parameters, but maximum stability is approximately the same, albeit with a different number of PP1 molecules.

The overall stability of the system is dependent on the lifetimes of both the UP and DOWN states. In general, any change in the system that increases the rate of phosphorylation tends to increase the lifetime of the UP state while reducing the lifetime of the DOWN state. The opposite is true for an increase in dephosphorylation rate. This tradeoff between lifetimes of the two states can be demonstrated by varying the number of phosphatase enzymes in the system while all other parameters are fixed. As seen in Figure 2C, a reduction in the amount of phosphatase resulted in an increased lifetime of the UP state, but destabilization of the DOWN state; in contrast, increasing the amount of phosphatase had the opposite effect (Figure 2D). Figure 2E (blue) shows the lifetimes averaged over 400 transitions, as a function of the number of phosphatase enzymes. Again, the lifetime of the UP state decreases, and the lifetime of the DOWN state increases with the number of PP1 molecules. We define the “system's lifetime” as the smaller of the two lifetimes, because we assume that a random potentiation of a synapse is as equally undesirable as a random depotentiation; a spontaneous transition from either state would be detrimental for memory. It follows that the system is optimal at the crossing of the two curves (Figure 2E), where the two lifetimes are equal (so that neither lifetime is too small). Such an optimum corresponds to a balance between the processes of phosphorylation and dephosphorylation. We therefore define the phosphatase concentration at which the two curves cross to achieve balance as the optimal concentration.

In the simulations described above, we set the optimal phosphatase concentration equal to the concentration of the CaMKII holoenzymes (R. J. Colbran, personal communication; see Table 1). We adjusted the less well-constrained parameters to achieve this. To see how sensitive the system's lifetime is to these particular choices, we simulated the system with a different value of the phosphorylation rate constant for the kinase, changing *k*_1_ from 1.5 s^−1^ to 0.75 s^−1^. Figure 2E (red) shows that the optimal phosphatase concentration is also reduced, but at this concentration the system lifetime remains as high as in the original system (the intersection of the two curves is shifted but remains at the same lifetime). We also tested the system's robustness to a 25-fold larger value, 10 μM, for *Κ_M_*. With an increased optimal phosphatase concentration, the system of 20 holoenzymes was stable for over 10 y. Thus, achieving long lifetimes does not require a specific level of enzyme activity, but does require an appropriate balance between phosphorylation and dephosphorylation rates. See Discussion for how this might be achieved.

### Stability Increases Exponentially with the Number of Molecules

We next considered how the system's lifetime varies with the number of holoenzymes (while PP1 varies in proportion, as does the system's volume). Figure 3A and 3B show the behavior of the model switch with four and 16 holoenzymes, respectively. We found (Figure 3C) that the system stability increases exponentially with the number of holoenzymes (i.e., the system size). As a result of this exponential dependence, the lifetime of the system almost doubles for each additional holoenzyme in the PSD. These simulations show that a switch made of only four holoenzymes can only be expected to have stability on the order of days to weeks, whereas increasing the system to 16 holoenzymes could result in a switch that is stable for a human lifetime.

**Figure 3:**
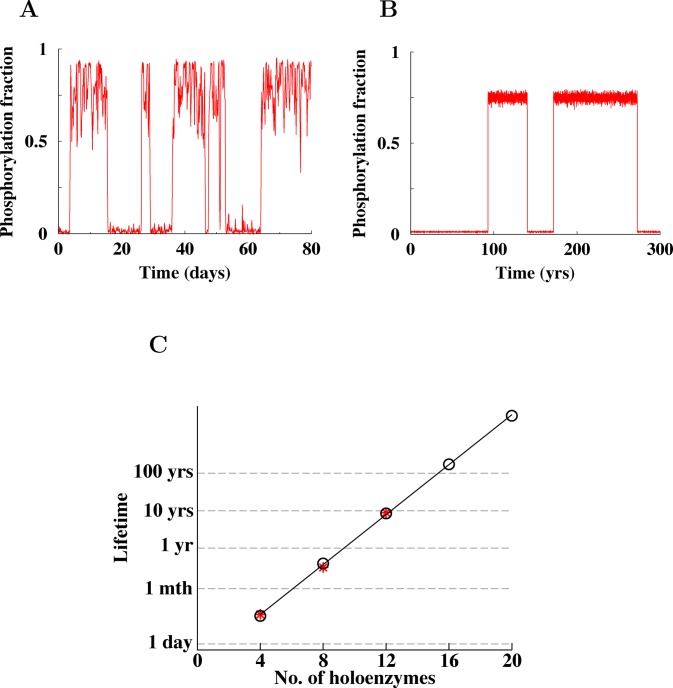
Stability of the Switch Increases Exponentially with System Size (Number of CaMKII Holoenzymes and PP1 Molecules) (A) Spontaneous transitions in a system with four CaMKII holoenzymes and four PP1 enzymes. Phosphorylation fraction is *Sp_tot_*/12*N_CaMK_*. (B) System with 16 holoenzymes and 16 PP1 enzymes, with four times the volume of (A). Note different timescales between (A) and (B). Phosphorylation fraction is *Sp_tot_*/12*N_CaMK_*. (C) The switch's lifetime increases exponentially with system size. Numbers of all species scale together with system volume. Circles are data points, line is a linear fit, indicating an exponential dependence, because the ordinate is in logarithmic scale. The red asterisks indicate data points where we included the PP1–I1P fluctuations explicitly.

### Protein Turnover Limits Memory Lifetime

In order to understand more deeply the cause of spontaneous switching, we examined what was occurring in the switch during the period preceding switching events. The two examples in Figure 4A and 4B show the total instantaneous level of phosphorylation of the system during the time preceding a spontaneous transition to the DOWN state (Figure 4A is for a system of four holoenzymes, Figure 4B for a system of 16). Holoenzyme turnover events are evident in these traces, because a turnover event causes an abrupt drop in the level of phosphorylation (marked by red arrows). In Figure 4A, four turnover events occur in a 3-h period prior to a downward switching event, and in Figure 4B, six turnover events occur in the same length of time (*t* = −7 h to −4 h), after which intrinsic dynamics take over to complete the downward transition. Based on the average time for turnover of 30 h per holoenzyme, one would expect a turnover event every 7.5 h in a system with four holoenzymes, and every 1.9 h in a system with 16 holoenzymes. Hence, the figures indicate that high numbers of turnover events occur in the periods before a transition, as expected if turnover initiates switching to the DOWN state (as explained on the next page). Such high amounts of turnover result from the stochastic nature of the turnover process, and occurred prior to such spontaneous transitions in all the traces we examined. Hence, protein turnover, and, in particular, its stochastic nature, strongly affects the system's ability to store information.

**Figure 4:**
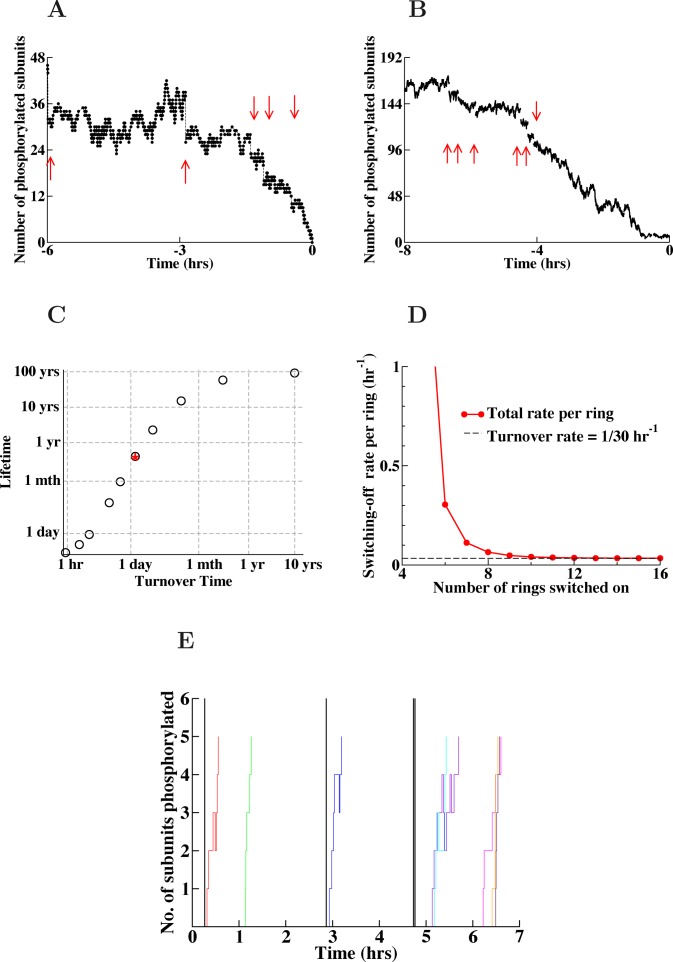
The Rate of Protein Turnover Limits the Maximal Lifetime of the System and Leads to a Minimal Rate of Energy-Consuming Activity (A and B) Stochastic changes in total phosphorylation during a transition from the UP to DOWN state, with turnover events marked by arrows, in a system with (A) four CaMKII holoenzymes and (B) 16 CaMKII holoenzymes. Red arrows indicate turnover events, which cause an abrupt drop in the level of phosphorylation. (C) Log–log plot of lifetime of the switch as a function of turnover rate for the system with eight CaMKII holoenzymes (the red asterisk marks 30-h turnover, used as standard in this paper). (D) The rate for an individual ring of subunits to switch off as a function of the total number of rings that are on (shown here for a system with eight CaMKII holoenzymes). As more rings are turned on, the phosphatase activity saturates and the equilibrium level of phosphorylation per ring increases. As a result, the switching-off rate for rings in the UP state for the system approaches the turnover rate (dashed line), because the probability of total ring dephosphorylation by PP1 becomes small. (E) Dynamic equilibrium between turnover (vertical solid black lines) and switching on of rings (colored step-like lines) when the system is in the UP state. At the time of the first turnover, five rings are already unphosphorylated by prior turnover. The system is stable because the rate of rings switching on matches the rate of turnover of phosphorylated rings (each turnover event can result in the loss of zero, one, or two phosphorylated rings). A system with 20 holoenzymes is shown.

We next investigated how the rate of protein turnover,*ν_T_,* affects the switch's stability. We found that an increase in the rate of protein turnover has little effect on the lifetime of the DOWN state, but dramatically reduces the lifetime of the UP state. Again, this is to be expected if turnover is responsible for initiating a switch DOWN in the system. Since the system is optimal when the two lifetimes are similar, in a series of simulations where we used different rates for protein turnover, we also adjusted the amount of PP1 to return to an optimal system (where UP and DOWN state lifetimes are similar). Hence, we can plot in Figure 4C the optimal lifetime of the switch as a function of average time for protein turnover. For turnover times of less than 1 mo, the optimal system stability is strongly dependent on the rate of turnover, suggesting that protein turnover is a limiting factor in the stability of the switch. Indeed, making the turnover rate very fast (hourly) can cause the system to lose all bistability.

In the UP state of our system, protein turnover replaces phosphorylated holoenzymes with unphosphorylated ones and is thus effectively acting like a phosphatase. It was therefore of interest to compare this effective phosphatase activity to the rate of dephosphorylation produced by the phosphatase itself. We proceeded by calculating the total rate for individual phosphorylated rings to switch off, that is, to become an unphosphorylated ring in state P0. Such a switching-off rate is the sum of the turnover rate and the rate for dephosphorylation by phosphatase. Our approximate calculation is accurate when the switching-on and switching-off rates for a ring are much slower than the rates for individual subunits to be phosphorylated or dephosphorylated, as it assumes a ring has time to reach all configurations of phosphorylation before it switches off (see Materials and Methods). Given that assumption, we obtained the proportion of time a ring that is on spends in each configuration of phosphorylation. The unphosphorylated state, P0, where a ring is off, can be reached either by turnover, or by dephosphorylation from the state with a single phosphorylated site, P1. Hence, the average rate at which a ring switches off (Figure 4D) is given by *ν*_3_ *Ρ_Ρ_*_1_ *+ ν_T_,* where *ν*_3_ is the rate per phosphorylated subunit of phosphatase activity (*ν*_3_ of equation 8), *Ρ_Ρ_*_1_ is the proportion of time a ring that is on spends in the configuration P1 and *ν_T_* is the protein turnover rate. Note that as the number of rings switched on increases, so the total phosphorylation of the system increases, causing both *ν*_3_ and *Ρ_Ρ_*_1_ to decrease. As is evident in Figure 4D, when more than half the rings in the system are on, the rate of rings switching off becomes identical to the turnover rate, *ν_T_,* itself (horizontal dashed line in Figure 4D). Hence, with a 30-h turnover rate and the optimal concentration of phosphatase, the phosphatase is unable to switch a ring off in the UP state; loss of phosphorylated rings is purely due to turnover.

We next sought to visualize how the system remains in the UP state even while turnover is causing the replacement of approximately two-thirds of the phosphorylated holoenzymes with unphosphorylated ones during a 30-h period (on average). In Figure 4E, we present a snapshot of a few hours of activity to show the time course for turnover and rephosphorylation of individual rings. The system in Figure 4E contains 20 holoenzymes, so the UP state is stable for many decades. Turnover events are marked by vertical lines. Unphosphorylated rings that become phosphorylated are indicated by the colored step-like lines, where each step indicates the phosphorylation of a subunit. It should be noted that at any one time, even though the system is in the UP state, a number of rings are unphosphorylated because of previous turnover. The rate of switching on for rings is proportional to the number of rings off. On average, the total rate of switching on matches the rate of phosphorylated rings lost by turnover. This dynamic equilibrium between the switching on of rings and turnover determines the average number of unphosphorylated rings at any time. In the system of 20 holoenzymes (and therefore 40 rings), the number of unphosphorylated rings typically varies from four to eight. The number is five before the first turnover event on Figure 4E and reaches a maximum of nine following the two closely spaced turnover events to the right in Figure 4E. The figure also illustrates how once one subunit is phosphorylated on a ring, the others rapidly follow.

### Effect of Fluctuations in Reactant Concentrations

In our simulations thus far, we have not considered noise in the signaling pathways that control the kinase and phosphatase reactions. A careful analysis of this issue requires an understanding of the signals that lead to bidirectional synaptic modification, as well as a consideration of the noise reduction mechanisms; both are beyond the scope of this paper. Nevertheless, we wanted to determine whether the switch we have modeled could tolerate reasonable noise levels in its input. In this class of models, reaction rates are nonlinear in Ca^2+^ concentration, so fluctuations in Ca^2+^ concentration affect the mean reaction rate, as well as providing additional noise about the mean rate. Moreover, the functional dependence on Ca^2+^ is not the same for all reaction steps. In particular, fluctuations in Ca^2+^ concentration increase the average rate of the slow initial (P0 to P1) phosphorylation step, which requires two Ca^2+^/calmodulins, to a greater extent than any other reaction steps. The change in relative reaction rates means that, in principle, large enough fluctuations can destroy the bistability altogether, whatever the system size. This class of switch has no absolute protection against Ca^2+^ fluctuations—indeed, we require in our model that a strong enough change in Ca^2+^, as occurs during LTP, leads to a switch from the DOWN to the UP state (see Figure 1C). However, the system should be robust to smaller, realistic fluctuations that occur in the absence of LTP. Figure 5A (blue squares) shows that the system with our standard parameters (but with a lower, 0.07 μM, baseline) is able to tolerate the moderate fluctuations that might arise from Ca^2+^ influx through NMDA-receptor-mediated channels (0.1 μM amplitude with 100 ms decay time; [44]) due to spontaneous presynaptic action potentials (a 0.5-Hz Poisson train) with only modest reduction in stability. Although the lifetime increases less steeply with system size than in the case without fluctuations (Figure 5A, black circles), extrapolation suggests that a system with 20 holoenzymes would be sufficient to have a lifetime of 100 y. If we use larger fluctuations, of amplitude 1.0 μM, it is important to adjust parameters to compensate for the change in average activity produced by fluctuations. With such an adjustment (see Materials and Methods) the system with 1.0-μM fluctuations in free Ca^2+^ concentration (with 100 ms decay time, above a 0.1**-**μM baseline in a 0.5-Hz Poisson train) is slightly more stable than the original system without fluctuating Ca^2+^ (Figure 5A, red triangles). We conclude that plausible levels of Ca^2+^ fluctuations in spines do not necessarily compromise switch stability.

**Figure 5:**
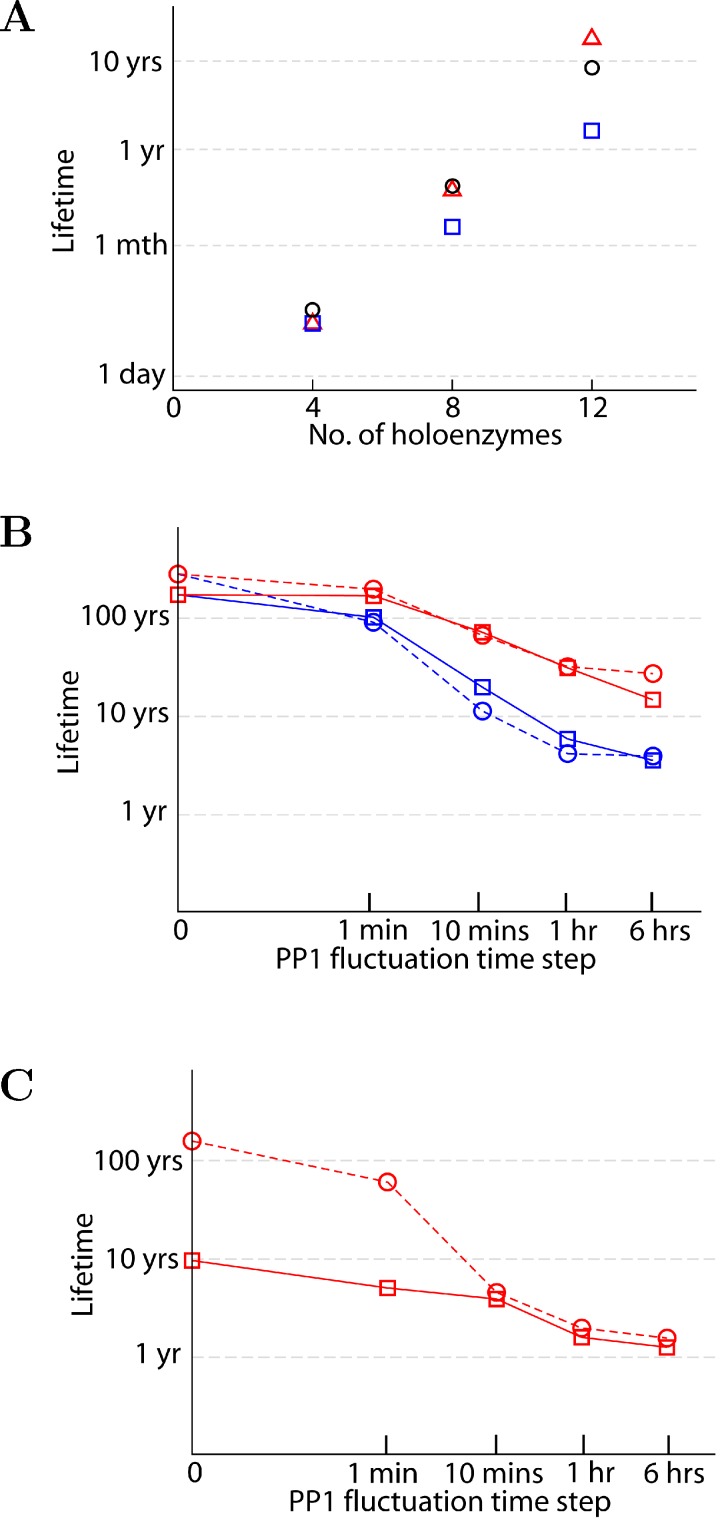
Switch Stability in the Presence of Spontaneous Fluctuations in Free Calcium Concentration and the Total Number of Enzymes (A) Effect of Ca^2+^ fluctuations on stability (lifetime) as a function of number of CaMKII holoenzymes. Circles indicate the original system without Ca^2+^ fluctuations. Squares indicate the original system with free Ca^2+^ fluctuations of amplitude 0.1 μM and baseline 0.07 μM. Triangles indicate adjusted system with free Ca^2+^ fluctuations of amplitude 1.0 μM and baseline 0.1 μM. The adjusted system has alternative parameters, such that *N_PP_*_1_ = *N_CaMK_*/2, *k*_1_ = 6 s^−1^, *k*_2_ = 7 s^−1^, and *K_H_*_1_ = 4.0 μM. The ordinate is in logarithmic scale. (B) Effect of fluctuations in the number of PP1 molecules on the lifetime of UP states (squares/solid line) and DOWN states (circles/dashed line) (16 holoenzymes). The timescale on the abcissa is the average time for the number of PP1 molecules to increase or decrease by one. Red indicates 12 < *N_PP_*_1_ < 20. Blue indicates 8 < *N_PP_*_1_ < 24. The ordinate is in logarithmic scale. (C) Lifetime of UP states (squares/solid line) and DOWN states (circles/dashed line) when the number of holoenzymes and PP1 molecules fluctuate in the respective ranges 14 < *N_CaMK_* < 18 and 8 < *N_PP_*_1_ < 24. The timescale for PP1 fluctuations varies along the abcissa. The timescale for CaMKII fluctuations is fixed by the turnover rate (30 h per holoenzyme). The ordinate is in logarithmic scale.

In our simulations so far, we have assumed fixed numbers of enzyme molecules, but in principle these numbers may fluctuate with time, potentially compromising stability. We implemented several sets of simulations to address this issue. In each set, we carried out a number of simulations corresponding to a range of timescales for fluctuations of PP1. Specifically, we varied the average time between random steps of plus one or minus one in the number of PP1 molecules in the PSD, and plotted this time as the x-axis in Figure 5B and 5C. It should be noted that the total time the system spends away from its optimum is usually the sum of many such time steps. In the first set of simulations, with the number of CaMKII holoenzymes held fixed, we assumed the number of PP1 molecules could fluctuate by plus or minus 25%, so in the particular case of 16 holoenzymes, the number of PP1 molecules varies between 12 and 20. The resulting lifetimes for the UP and DOWN states are given in Figure 5B in red. The lifetime decreases as the average time between changes in PP1 increases. This is because slow fluctuations lead to long periods of time when the system is far from optimal. In contrast, if the fluctuations are rapid, the system may not have time to make a transition even if the system loses bistability temporarily. Still, even with a change every 6 h, the system with 16 holoenzymes is stable for over about 20 y. Second, we increased the amplitude of fluctuations in PP1 to plus or minus 50% (a variation of a factor of three, from eight to 24 in this case). The simulation results in Figure 5B (blue) indicate that such an increase in amplitude of fluctuation causes the average lifetime for the system to decrease. Again, if the fluctuations are relatively rapid, they do not seriously degrade the switch. It is somewhat remarkable that when the number of PP1 molecules varies by a factor of three over a timescale of tens of minutes, the switch lifetime still averages over 10 y. Third, we introduced slow fluctuations in the number of CaMKII holoenzymes, assuming stochastic insertion of holoenzymes as well as stochastic turnover. Since the timescale of removal is set at 30 h per holoenzyme [35], the average rate of insertion is fixed (at 16 every 30 h) to ensure the appropriate average of 16 holoenzymes within the PSD. As above, the simulations covered a range of timescales for the fluctuations of PP1, whose number could vary between 8 and 24. We found that variations in the number of holoenzymes are more deleterious than variations in PP1 alone. In particular, loss of holoenzymes from the PSD destabilizes the UP state. This is because the effect seen above, of a nonoptimal CaMKII to PP1 ratio, is exacerbated by a reduction in switch size when holoenzymes are lost (cf. Figure 3C). Figure 5C indicates the resulting lifetimes when the number of CaMKII holoenzymes varied between 14 and 18. Without PP1 fluctuations (equivalent to a time step of zero) the DOWN state is little affected by these slow fluctuations in the number of holoenzymes (circles/dashed line), but stability of the UP state is greatly reduced (squares/solid line). Including slow PP1 fluctuations of **±**50% reduces the lifetimes of both UP and DOWN states to below 10 y. Although a system averaging 20 holoenzymes would be more stable, we conclude that during turnover, a holoenzyme removed from the PSD needs to be replaced relatively rapidly—on a timescale of minutes, not hours—to avoid degradation of the switch. Moreover, if CaMKII were not anchored, but able to freely diffuse in and out of the PSD, fluctuations in the number of holoenzymes present would be increased, and bistability would not be possible [45,46].

## Discussion

In this paper, we have considered the stability against fluctuations of a bistable switch based on the interaction of CaMKII and PP1 in the PSD. Although a deterministic model of such a switch has been presented before [28,29], it was not previously possible to assess quantitatively the potentiality of the switch for long-term information storage, because the rate of spontaneous reset was not known. Given the small number of CaMKII molecules at synapses [32], stochastic fluctuations in the reactions must necessarily lead to switch reset on some timescale. Our results show that that this timescale depends crucially on the number of molecules that make up the switch (see Figure 3C). Indeed, this dependence is highly nonlinear, scaling exponentially with the number of molecules involved. We have shown that this timescale can exceed human lifetimes when the number of holoenzymes is greater than 15 (see Figure 3B), provided the parameters of the system are in an optimal range. A substantially smaller number of holoenzymes, such as four, would result in spontaneous transitions on a timescale of a week (see Figure 3A). One interesting possibility is that initially a small number of CaMKII holoenzymes is sufficient for the immediate information storage and with time, during an initial consolidation period [47,48], a larger number of holoenzymes accumulate at the PSD, allowing for more permanent memory formation (see Figure 3C). Our general conclusion is that relatively small groups of CaMKII molecules, such as are found in the PSD (where the average is 30 holoenzymes) can function as highly stable switches and could potentially subserve information storage for very long periods.

The exponential dependence of lifetime on system size is consistent with general theoretical considerations [11]. This is because the switch can be described as a biochemical system with a “double well” effective energy potential in which two minima are separated by a barrier. The fluctuations in the reactions generate noise-driven hopping over the barrier in a manner analogous to thermally driven hopping over a real potential barrier [11,49,50,51]. The effective barrier height is proportional to the system size [49], and it is well-known that with a constant noise source, the time for transitions across a barrier increases exponentially with barrier height [51,52]. Intuitively, a transition from the UP to DOWN state is triggered when a critical number of CaMKII rings (proportional to the system's size *N*) are dephosphorylated at the same time. In terms of probability, the decrease in likelihood with *N* is the same effect as the increase in expected number of coin tosses necessary to obtain *N* heads in a row, each with a probability *p* = 1/2. The probability for *N* consecutive heads is *p^N^*, and the expected time (number of coin tosses) it takes before this happens is (1/*p*)*^N^* = 2*^N^,* which grows exponentially with *N*. By analogy, the larger the system, the more rings of CaMKII have to turn off randomly without recovery before they are able to cause a switch in the whole system. If the dephosphorylation events do not occur “in a row,” switching off a critical number of CaMKII rings within a short time interval, the opposing reactions (autophosphorylation) have time to counteract and turn rings on, allowing the natural dynamics to drive the system back to the UP equilibrium state.

An additional finding is that the molecular turnover of CaMKII strongly limits switch stability (see Figure 4). If turnover were absent, switch stability could be an order of magnitude higher (see Figure 4C). Our analysis shows an interesting set of relationships between the rates of phosphatase and kinase activities and the rate of protein turnover with regards to their effect on switch stability. In principle, lowering the basal phosphatase and kinase rates in proportion increases switch stability. Such lowering of the dephosphorylation rate has a second desirable feature of lowering energy consumption. This is because the UP state consists of what biochemists call a “futile cycle,” in which the rate of ATP-utilizing phosphorylation equals the rate of dephosphorylation. Although minimizing energy utilization dictates that the system be “cooled” (lowering the phosphatase and kinase rates), our results show that there are limits to how much cooling is effective and that this limit is set by the protein turnover rate. Specifically, if cooling sets the phosphatase and kinase rates too low, the system cannot regain steady-state values after a molecular turnover event (newly inserted unphosphorylated kinase molecules will not become fully phosphorylated, as they do in Figure 4E). In this case, unphosphorylated kinase molecules will accumulate, leading inexorably toward the threshold for reset to the DOWN state.

Our results suggest that the stability of a switch depends sensitively on a balance of phosphorylation and dephosphorylation rates. Hence we assessed how changes in the ratio of PP1 to CaMKII affect the lifetimes of states of the switch (see Figure 2E). We find that short-term fluctuations in the ratio, on a timescale of tens of minutes, do not significantly degrade the switch (see Figure 5B). Slower fluctuations are more deleterious, particularly as stability of the UP state is compromised if the number of CaMKII holoenzymes becomes too low (see Figure 5C). In contrast to its robustness to short-term fluctuations, our system requires the long-term average ratio of activities is constrained (see Figure 2E). Hence, if all other parameters and concentrations are fixed, the ratio of numbers of PP1 molecules to CaMKII molecules should lie in a narrow optimal range (see Figure 2E). It will thus be of interest to see whether special mechanisms exist to stabilize the ratio of PP1 to CaMKII in the long term. Promoting the necessary fixed ratio of PP1 to CaMKII may be one of the functions of the scaffolding proteins that hold CaMKII and PP1 within the PSD structure [53,54,55,56,57,58]. Moreover, our results (see Figure 5A) indicate that moderate Ca^2+^ fluctuations are tolerable on short timescales, but we find the average level must be tightly regulated over the long term (to within several percent; data not shown). Since the kinase activity depends on the level of free calmodulin, it may further be expected that free calmodulin is regulated over long timescales. This may be an important function of the known calmodulin buffers [59,60]. In the absence of control mechanisms, the stability of the switch would be greatly reduced.

Several limitations of our study should be noted. While we addressed the effect of Ca^2+^ fluctuations (see Figure 5A), we did not include the detailed reaction steps of Ca^2+^ binding to calmodulin in our model, but used the steady-state values for reaction rates based on Ca^2+^/calmodulin. These steady-state rates may not be reached during rapid changes in concentration. Hence, the phosphorylation rates may not vary with Ca^2+^ precisely as we have modeled, in which case other parameters or concentrations would have to be altered to maintain an optimal system. However, including calmodulin-binding steps, and removing the assumption of excess, freely available calmodulin, would reduce the effect of sharp, brief rises in free Ca^2+^. Hence, the influx of Ca^2+^ necessary to cause LTP could be greater than presented here (see Figure 1C), and the system may be stable to larger Ca^2+^ fluctuations than those we include. Quantitative measurements of spontaneous Ca^2+^ fluctuations in vivo will be needed to assess whether the switch stability is robust against realistic fluctuations. A second type of simplification that we have made is likely to lead to an underestimate of stability. We have assumed that the CaMKII molecules bound in the PSD are operating with the same kinetic constants measured in free solution. However, some or all of the CaMKII in the PSD may be bound to NMDA receptors [61,62]. This binding increases the rate of autophosphorylation of the first site, allowing it to occur with only one calmodulin bound rather than two. If such binding to NMDA receptors occurs significantly only after an LTP event, when the system is in the UP state, the effect of protein turnover will be reduced, because unphosphorylated rings would become rapidly phosphorylated before the whole system has time to switch to the DOWN state.

While the switch we have described could be stable for a human lifetime, long-term information storage may not require stability of this magnitude. One possibility is that such long-term stability is not solely a property of the switch, but emerges from interaction between the switch and an attractor network created by memory-specific synaptic connections. According to this idea [47,48], reactivation of the attractor, perhaps during sleep, may serve to refresh the memory by setting switches back to their correct state. For such a system to work, average switch stability need only be larger than the time between reactivations of the attractor. Such reactivations appear to be important in the early stages of memory, when consolidation is important [47,63]. However, we know little quantitatively of how frequently such reactivation processes take place. Moreover, the role of reactivation in long-term maintenance of a memory trace, after initial consolidation, remains unclear. Advances in this direction would enhance our understanding of the interplay of molecular and network properties in determining the overall stability of memory in the brain.

## Materials and Methods

### 

#### The model

We treat each ring of six subunits of CaMKII as an independent entity [37,64,65,66]. Each subunit can be in one of two states: either phosphorylated or unphosphorylated. The set of possible configurations among the six subunits results in 14 distinguishable states for a ring, labeled here by the number of phosphorylated subunits: P0, P1, P2 (three configurations for P2 because the two phosphorylated subunits can be either neighboring, or separated by either one or two unphosphorylated subunits), P3 (four configurations), P4 (three configurations), P5, and P6. The different configurations have different multiplicities, which are counted when calculating rates of reactions that change configurations. Phosphorylation of the first subunit of a ring (P0 to P1) requires the binding of two activated calmodulins, so is slow at resting Ca^2+^ concentrations (see Figure 1A). Once a single subunit is phosphorylated, it can catalyze the phosphorylation of neighboring subunits in a directional manner [27], so that further phosphorylation steps are faster at resting Ca^2+^ concentrations (see Figure 1A). We refer to a ring in the unphosphorylated state, P0, as off, and a ring with any other level of phosphorylation as on. Once a ring is on, we do not include further slow steps for that ring in the calculations, because the faster, directional steps dominate.

Taking a Hill coefficient of three [40,67], we have for the initial phosphorylation rate per subunit:



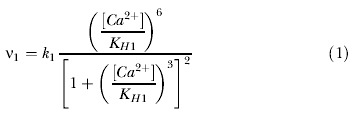



and for the phosphorylation of a clockwise neighboring subunit:



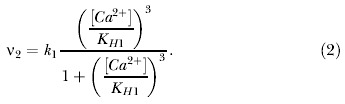



The dephosphorylation occurs through the Michaelis–Menten scheme:



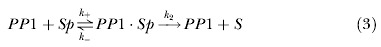



with Michaelis constant, 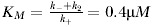 , and where *Sp* denotes a phosphorylated subunit while *S* denotes an unphosphorylated one. In the simulations presented here, we assume *k*_+_ = *k*_2_/*K_M_* and set *k*_−_ to zero, but we find the results are not noticeably influenced by the relative values of *k*_−_ and *k*_+_ at fixed *k*_2_ and *K_M_*.

The phosphatase is deactivated by phosphorylated inhibitor-1 (I1P), a noncompetitive antagonist [68,69,70]. We follow the formulation of Zhabotinsky [28], assuming the level of free inhibitor-1 (I1) is constant (at 0.1 μM) in the PSD owing to free exchange of I1 with the larger cell volume. Such free exchange with the larger cell is important, as the number of free I1 molecules in the PSD is less than one on average. Even the spine itself can contain fewer I1 molecules than there are PP1 molecules in the PSD. However, the rapid and strong binding of PP1 to the inhibitor means that the PSD acts as a sink of free I1, and the total concentration of all I1 in the PSD is significantly greater than that of free I1. Importantly, I1 exchanges between the PSD and spine volume vary rapidly, and between the spine and parent dendrite with τ ≤ 1 s, a timescale much faster than that of the phosphatase and kinase reactions.

I1 is phosphorylated by cAMP-dependent protein kinase and dephosphorylated by calcineurin. The rate of dephosphorylation of I1P by calcineurin increases with Ca^2+^, with a Hill coefficient of three [71], because calcineurin requires Ca^2+^/calmodulin to activate. Hence I1 is less phosphorylated and the phosphatase is less inhibited at higher Ca^2+^ concentrations. These reaction steps (modeled by Zhabotinsky [28]) are assumed to be fast, so we can write down the stationary level of free I1P concentration as



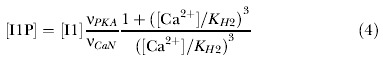



and the fraction, *f_e_,* of phosphatase that is free of inhibitor and hence active as



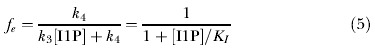



with *K_I_ = k*_4_ */k*_3_.

In the majority of results presented here, we do not simulate the reaction of phosphatase inhibition stochastically. Since the reaction occurs on a timescale faster than other reaction steps, we can use the quasi-steady-state assumption and use only its equilibrium values [72]. We did test this assumption by carrying out simulations that included both a stochastic step for phosphorylation of I1 (instead of equation 4) and for inhibition of PP1 (instead of equation 5). Simulating such fast processes (two to three orders of magnitude faster than other reactions) means a huge increase in the total number of reactions per unit time and hence a corresponding increase in the computer time required. The results for the three systems we tested (red asterisks in Figure 3C) showed no significant difference from the results without such fast fluctuations. Hence, we have confidence in our use of the equilibrium values for other data points.

Turnover occurs at a rate, *ν_T_,* and acts equivalently to a non-saturating dephosphorylation process, as we assume all holoenzymes are replaced by unphosphorylated ones, and any attached PP1 is released back to the system.

It is possible to calculate analytically *ν*_3_, the rate of dephosphorylation by PP1 per phosphorylated subunit. Note that from Figure 1B, we require *ν*_3_ to decrease at high phosphorylation so that the total concentration of subunits dephosphorylated per unit time, *ν*_3_·[*Sp_tot_*], saturates (we have written the total concentration of phosphorylated subunits, [*Sp_tot_*] = [*Sp*] + [*PP*1.*Sp*]). To write the rate of dephosphorylation on the right hand side of equation 3 as *ν*_3_·[*Sp_tot_*], we assume the intermediate product, [*PP*1.*Sp*], is at steady state. We combine equation 3 with the other two ways that [*PP*1.*Sp*] can change. That is, noncompetitive inhibition leads to the reaction



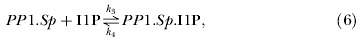



while turnover leads to



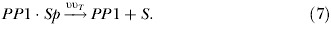



So we calculate an effective dephosphorylation rate constant, *ν*_3_, from *ν*_3_·[*Sp_tot_*] = *k*_2_[*PP*1.*Sp*] assuming *d*[*PP*1.*Sp*]/*dt =* 0. The result is, writing [*E*_0_] = *N_PP_*_1_ *N_A_*/*vol,*



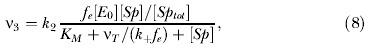



where the concentration of phosphorylated subunits without phosphatase attached, [*Sp*], is given by



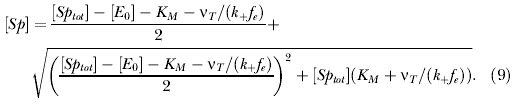



As expected, the rate, *ν*_3_, decreases significantly for [*Sp_tot_*] > *K_M_* such that at large total phosphorylation in the UP state, the product 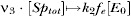 is a constant (see Figure 1B). In the limit of negligible phosphorylation, 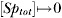 , equation 9 simplifies to give



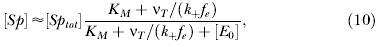



hence, from equation 8,



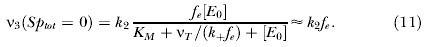



In the limit of high phosphorylation (where 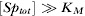 ), equation 9 becomes



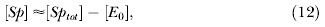



leading to



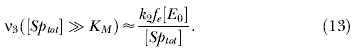



Our derivation differs from the standard Michaelis–Menten approach because we cannot assume that 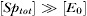 at all times, so [*Sp*] ≠ [*Sp_tot_*].

#### Monte Carlo simulations

We conducted Monte Carlo simulations of this model, in which all the microscopic configurations of CaMKII holoenzymes were counted and chemical reactions between these states were simulated as stochastic Markov processes. We used the algorithm of Gillespie [51,73], which can be summarized as follows. We identify all the possible configurations of rings. There are 56 in total, because for a configuration with a given number, *n,* of phosphate groups, the number of PP1 bound can vary from 0 to *n* (assuming *n* < *N_PP_*_1_). So for each of the 14 configurations of phosphate groups, the number of configurations including enzymes is multiplied by *n +* 1. For example, a ring with two subunits phosphorylated (P2) can have up to two PP1 enzymes attached. As there are three distinguishable configurations for two phosphorylated subunits on a ring, and each configuration can have zero, one, or two PP1 enzymes attached, we include nine separate configurations for P2. We have the following numbers of configurations: P0 (1), P1 (1 × 2), P2 (3 × 3), P3 (4 × 4), P4 (3 × 5), P5 (1 × 6), and P6 (1 × 7), to obtain 56 in total. We do not explicitly count the different relative positions of PP1 bound to phosphate groups, but just select one of the phosphorylated subunits at random when the dephosphorylation occurs.

At any point in time, the system is in a state that is defined by the number of rings in each configuration, denoted by {*N_i_*}, *i* = 1, 2,…, 56. The numbers of rings in each configuration {*N_i_*} determine the rates of each of the possible reaction steps, {*r_j_*}. A reaction step takes a ring from one of the 56 configurations to another. With the three types of reaction steps (phosphorylation, PP1 binding, or dephosphorylation) able to occur from most of the configurations, in total the system has 144 distinguishable reaction steps. However, most of the rates are zero at any particular instance. Protein turnover is treated stochastically like any other process. Turnover results in two randomly chosen rings (i.e., one holoenzyme) being replaced by two rings in the state P0 (totally unphosphorylated).

The fundamental assumption behind the simulations is that any particular rate, *r_j_*(*t*), depends only on the present configuration, not on the history of the system. This makes the reaction scheme a Markov process. The key step that is necessary when dealing with small numbers of molecules is to recall that the deterministic rate is the macroscopic average of a stochastic process, such that the probability, *P_j_dt* of reaction step *j* in a small time interval, *dt,* is given by



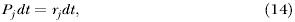



that is, the rate is simply the probability of occurrence per unit time, and, given the Markov assumption, we now have a Poisson process.

Gillespie's algorithm proceeds by first summing the rates of all reaction steps to obtain the average rate, *R_T_,* for any change in configuration:



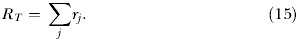



The distribution of times elapsed before a reaction step follows an exponential decay,



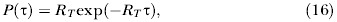



which is a standard result, easily verified as it satisfies the necessary requirement:



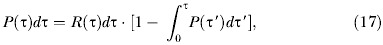



that is, the probability of reaction in time step from *τ* to *τ* + *dτ* equals *R*(*τ*)*dτ* multiplied by the probability that a reaction did not happen before *τ*.

Once the time for the next reaction step is randomly selected, a second random selection is taken to decide which particular reaction occurs. The relative probabilities, *p_j_*, of each reaction step are simply proportional to their rates, *p_j_* = *r_j_*/*R_T_*.

Given the time and type of the next reaction step, the total time for the system is advanced by *τ*, and the quantities, {*N_i_*}, in relevant configurations are updated, as are the reaction rates for the affected reaction steps. The process now repeats itself with a new set of reaction rates. Total phosphorylation is monitored, and thresholds for determining a switch to the UP state or a switch to the DOWN state are set according to the equilibrium values, but typically if the total falls to below 10% phosphorylation, we record a transition to the DOWN state, and if it spontaneously rises to 70% phosphorylation, we record a transition to the UP state. It should be noted that the time it takes for the system to switch between UP and DOWN states is on the order of hours, which gives an overestimate of the lifetime of a state when in reality the system is “in transition.” However, such an error on the order of hours is insignificant (compared to many years for typical average lifetimes) except in the most unstable systems presented here.

Simulations begin with either 0% or 100% phosphorylation, but within a very short time (at least compared to the lifetimes of UP and DOWN states) the system settles near the equilibrium values for UP or DOWN states, respectively.

#### Switching-off rate calculations

We define a ring in the unphosphorylated state, P0, as off, and one with any subunits phosphorylated as on. We calculate the rate for rings to switch off by assuming that all rings in the system that are on reach a dynamic equilibrium in their phosphorylation levels, in the time between switching-on and switching-off events for rings. This approximation, known as separation of timescales, is valid when the individual subunit phosphorylation and dephosphorylation rates are much faster than the rates for rings to switch on and off (as evident in Figure 4E).

For a given amount of total phosphorylation, we know exactly the average activity of the phosphatase. Knowing this activity means average rates can be calculated for all reactions. The different average rates determine the rate of change from one configuration of a holoenzyme to another, so knowing the average rates allows us to obtain numerically the relative amounts of time, *Ρ_i_,* spent in each configuration, *i*. Hence we can calculate when the system has a given total level of phosphorylation, *Sp_tot_*, what is the average phosphorylation level per ring, 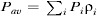 , where *P_i_* is the phosphorylation of configuration *i*. The total level of phosphorylation, *Sp_tot_*, when a given number of rings are on, *N_on_,* is given by



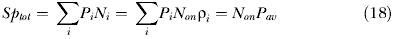



Since *Sp_tot_* is proportional to *P_av_* and *P_av_* depends on *Sp_tot_*, we iterate the equations to find a self-consistent solution for *Sp_tot_* and *P_av_* for each value of *N_on_*.

To find the switching-off rate per ring, plotted in Figure 4D, for each number of rings switched on, the total phosphorylation, *Sp_tot_,* is calculated as above. The value of *Sp_tot_* determines the phosphatase activity per subunit, *ν*_3_, which affects the proportion of time spent by a ring in each configuration. Notably, the lower *ν*_3_ is, the more phosphorylated are the on rings, and the lower is *Ρ_P_*_1_. The rate for a ring to switch off is the sum of the turnover rate, *ν_T_*, and rate of dephosphorylation by PP1 of holoenzymes with only a single phosphorylated subunit, *ν_3_Ρ_P_*_1_.

#### Ca^2+^ influx and fluctuations

For the LTP induction protocol, we use a burst of Ca^2+^ influx constituted by a Poisson train of Ca^2+^ pulses at 100 Hz. Each pulse is a 0.1-μM step increase, followed by a 100 ms exponential decay in free Ca^2+^ concentration.

In the study of the effects of background Ca^2+^ fluctuations, we assume Ca^2+^ entry through NMDA receptors occurs as a random Poisson train with an average rate of 0.5 Hz. We model each Ca^2+^ influx as a step rise of 0.1 or 1.0 μM, followed by exponential decay with a time constant of 100 ms ([44], assuming a membrane potential of −50 mV and a spine volume of 0.1 μm^3^; peak [Ca^2+^] rise is 0.14 μM per presynaptic spike). We reduce the base level of Ca^2+^ to 0.07 μM in the 0.1**-**μM amplitude simulations, keeping all other parameters the same, to maintain an approximate balance between phosphorylation and dephosphorylation rates. We assume a refractory period of 2 ms for the presynaptic neuron, so that no two Ca^2+^ influxes occur within such a short interval (hence the train of Ca^2+^ inputs is not quite Poisson, but includes a 2-ms negative correlation).

If the fluctuation amplitude is too large (for example, if it is doubled from 0.1 μM to 0.2 μM in this case) and the model parameters are fixed, then bistability is completely lost, because the fluctuations actually change the average effective kinetic rates of reaction steps and bring them outside of the range for bistability of the system. Hence, in the simulations with amplitude 1.0 μM, we maintain a base Ca^2+^ level of 0.1 μM and adjust other parameters (see legend of Figure 5) to maintain an appropriate balance between the different reaction rates. For example, we include a higher *Κ_H_*_1_ to maintain low rates for the initial phosphorylation step. With the alternative parameters, the system is only bistable if the Ca^2+^ fluctuations are present.

Notice that the number of free Ca^2+^ ions in the PSD is on average less than one. However, Ca^2+^ acts through Ca^2+^-bound calmodulin (which can be at a higher concentration), and the exchange of both free Ca^2+^ and calmodulin between postsynaptic density and the spine is much more rapid than both the fluctuations considered here and typical CaMKII reaction steps. Hence, we can neglect such strong, but fast, “shot” noise. Exchange of free Ca^2+^ or calmodulin between the dendritic shaft and spine will cause significant additional fluctuations, but only if it is on a slower timescale than the dissociation steps between Ca^2+^ and calmodulin, or Ca^2+^/calmodulin and CaMKII. Future work, including on these specific binding reaction steps [74], is necessary to clarify more precisely the dynamical effects of Ca^2+^ changes on the system.

#### Parameters

Parameters for the standard system are given in Table 1. In figures where one parameter varies, all others are fixed according to the table, unless otherwise stated. Figures where the number of holoenzymes changes also include a proportionally changed volume and number of phosphatases, to maintain fixed concentrations. The standard concentration of PP1 is equal to that of CaMKII holoenzymes, at 20 molecules per 10^6^ nm^3^ or 33 μM. Our maximal system with 20 holoenzymes is slightly smaller than an average synapse of 30 holoenzymes [32]. Hence, the volume is smaller than average, corresponding to a cylindrical PSD of diameter 250 nm (compared to an average diameter of 350 nm in [32]), assuming the holoenzymes are predominantly in a single layer of a little over 20 nm thickness, to give a volume of 10^6^ nm^3^ for the domain of reactions.

For those parameters in the table without experimental references, we chose values that were in a reasonable range given the values for similar chemical reactions. We picked simple values that would work well for our system. For example, a low Michaelis constant is beneficial because the range of bistability increases [28]. In general, variation of any one parameter, without alteration of the others, leads to an effect like that shown in Figure 2, where number of phosphatases is varied. If, for example, the activity of calcineurin is higher (e.g, giving *ν*_Ca*N*_= 2.0 instead of *ν*_Ca*N*_=1.0), then the amount of I1P is halved and the amount of uninhibited PP1 increases. Hence, the stability of the UP state decreases while stability of the DOWN state increases. However, a system with a lower overall PP1 concentration would work as well as the original system. So, modification of individual parameters does degrade the system, reducing the lifetime of one state relative to the other. Nevertheless, if the cell is able to maintain concentrations of species in an optimal range determined by the actual value of parameters, then the best results shown here can be achieved (cf. [28]).

## Abbreviations

CaMKII: calcium/calmodulin-dependent protein kinase II
I1: free inhibitor-1
I1P: phosphorylated inhibitor-1
LTP: long-term potentiation
NMDA: N-methyl-D-aspartate
PP1: protein phosphatase-1
PSD: postsynaptic density
*Sp_tot_*: total number of phosphorylated subunits
